# Identification and characterization of the fibrinogen-like domain of fibrinogen-related proteins in the mosquito, *Anopheles gambiae*, and the fruitfly, *Drosophila melanogaster*, genomes

**DOI:** 10.1186/1471-2164-6-114

**Published:** 2005-09-08

**Authors:** Xinguo Wang, Qin Zhao, Bruce M Christensen

**Affiliations:** 1 Department of Animal Health and Biomedical Sciences, University of Wisconsin-Madison, 1656 Linden Dr., Madison, WI 53706, USA; 2 Department of Biochemistry, University of Wisconsin-Madison, 433 Babcock Drive Madison, WI 53706, USA; 3 Promega Corp., 2800 Woods Hollow Road, Madison, WI 53711, USA

## Abstract

**Background:**

The fibrinogen-like (FBG) domain, which consists of approximately 200 amino acid residues, has high sequence similarity to the C-terminal halves of fibrinogen β and γ chains. Fibrinogen-related proteins (FREPs), which contain FBG domains in their C-terminal region, are found universally in vertebrates and invertebrates. In invertebrates, FREPs are involved in immune responses and other aspects of physiology. To understand the complexity of this family in insects, we analyzed FREPs in the mosquito genome and made comparisons to FREPs in the fruitfly genome.

**Results:**

By using the genome data of the mosquito, *Anopheles gambiae*, 53 FREPs were identified, whereas only 20 members were found in the *Drosophila melanogaster *genome. Using sequence profile analysis, we found that FBG domains have high sequence similarity and are highly conserved throughout the FBG domain region. By secondary structure analysis and comparison, the FBG domains of FREPs are predicted to function in recognition of carbohydrates and their derivatives on the surface of microorganisms in innate immunity.

**Conclusion:**

Detailed sequence and structural analysis discloses that the FREP family contains FBG domains that have high sequence similarity in the *A. gambiae *genome. Expansion of the FREP family in mosquitoes during evolutionary history is mainly accounted for by a major expansion of the FBG domain architecture. The characterization of the FBG domains in the FREP family is likely to aid in the experimental analysis of the ability of mosquitoes to recognize parasites in innate immunity and physiologies associated with blood feeding.

## Background

In mammals, fibrinogen, a soluble plasma protein, contains six polypeptide chains, two each of the Aα, Bβ and γ chains, linked by 29 disulfide bonds. Fibrinogen participates in both the cellular phase and the fluid phase of coagulation [1]. The fibrinogen-like (FBG) domain, which consists of approximately 200aa residues and has high similarity to the C-terminal halves of fibrinogen β and γ chains, has been found in a growing number of proteins [2]. Three distinct fibrinogen-related proteins (FREPs) have been identified in human: ficolin, tenascins, and microfibril-associated protein (MAP) [3-5]. These FREPs all contain a common C-terminal FBG domain with high sequence identity to the C-terminal regions of fibrinogen β and γ chains, but differ in their N-terminal regions. The FBG domain in ficolin can be brought together as clusters of three by collagen O-like triple helices, and is responsible for *N*-acetylglucosamine (GlcNAc) binding activity [6]. Recent studies have shown that human serum ficolins act as phagocytic receptors on circulating monocytes for microorganism recognition [7]. Tenascins are a family of multifunctional extracellular matrix (ECM) glycoproteins subject to complex spatial and temporal patterns of expression in the course of various organogenetic processes. These proteins mediate cell adhesion and show tissue-specific and cell growth-associated expression [4]. Microfibril-associated protein, another extracellular matrix protein, is a component of connective tissue microfibrils and a candidate for involvement in the etiology of inherited connective tissue diseases, which are associated with the Smith-magenis syndrome, a multiple congenital anomaly/mental retardation syndrome [8].

In invertebrates, several FREPs have been reported in various species, such as tachylectins from the horseshoe crab, *Tachypleus tridentatus *[9], fibrinogen-related proteins (FREP) from the snail, *Biomphalaria glabrata *[10], ficolins from the solitary ascidian, *Halocynthia roretzi *[11], tachylectin-related protein in the sponge, *Suerites domuncula *[12] and aslectin (AL-1) from the mosquito, *Armigeres subalbatus *[13]. All of these FREPs contain a common C-terminal FBG domain with high sequence identity to that of fibrinogen β and γ chains, but which differs in their N-terminal regions. These FREPs likely play an important role in the innate immune response against parasites [9,12,13]. The FBG domain of tachylectin is able to bind GlcNAc [9]. Aslectin, which also binds GlcNAc, is able to bind bacteria, and is likely involved in the antibacterial immune response in mosquitoes [13].

The rapid progress in the development of whole genome and expressed sequence tag (EST) databases provides an abundance of sequence data that greatly facilitates gene function studies. Using bioinformatics, one can mine the information from these databases to acquire an overview of each gene family and assess evolutionary relationships among its members [14]. Although the FREP family in the genomes of *Anopheles gambiae *and *Drosophila melanogaster *was briefly compared earlier [15], the FBG domains in this gene family have not been comparatively characterized. In this study, data derived from the genome and EST databases of the mosquito, *A. gambiae*, and the fruitfly, *D. melanogaster*, are presented here as an initial, yet exhaustive search for FREPs in both species. Provided is an overview of this protein family, including sequence alignments, patterns of conservation, and phylogenetic relationships. A further comparison between the annotated gene products from the genome sequences and the actual transcripts from the EST database also is made. In summary, these studies provide the first encompassing description of the FREP gene family in insects and establish a foundation for future studies that aim to define the role of these genes.

## Results and discussion

### Identification of FREP genes and characterization of the FBG domain in the *A. gambiae *genome

To identify FREP proteins encoded in the *A. gambiae *genome, a PSI-BLAST search was performed using AL-1 as a query sequence to screen the *A. gambiae *genome database at NCBI. Sixty amino acids were used as the minimum length of homology, and protein sequences having 35% or greater amino acid identity with AL-1 were added to the gene family list. To find FREPs that may have been overlooked due to low sequence identity to AL-1, we selected each sequence from the search results as a new seed to search the *A. gambiae *genome database again. Additional sequences were identified as homologs of the queries and added to the original list. This gene family list was manually examined to eliminate redundant sequences generated by repeated searching. This search revealed the presence of 53 genes encoding hypothetical FREP proteins in the *A. gambiae *genome (Table 1).

**Table 1:** Fibrinogen-related proteins in *A. gambiae *and *D. melanogaster*

Gene ID	Length (aa)^1^		FBG domain^2^	Chromosomal location	Transcription^3^	
				
	P	M			EST	cDNA library
***A. gambiae***						
EAA10385	201		full	2L 20D	-	
EAA10406	217		full	2L 20D	-	
EAA04425	186		full	2L 26A	+	Hemocyte
EAA10466	865	848	3' truncated	2L 21A	+	Development
EAA14231	226		full	3R 35B	+	NAP1
EAA44096	190		full	2L 23B	+	NAP1,NAH, Blood1,NAFB
EAA05203	296	273	full	3L 42B	-	
EAA05102	363	341	full	3L 42A	+	4A3A,NAP1,NAH, Blood1,NAFB
EAA05205	308		full	3L 42A	-	
EAA05224	310		full	3L 42A	+	4A3B, NAH, Blood1
EAA43404	314	292	full	3R 33C	-	
EAA01903	236		full	Unknown	+	NAP1
EAL39348	202		full	3L 40A	-	
EAA10360	688	660	full	2L 21A	-	
EAA00222	173		full	Unknown	-	
EAA13725	182		full	3L 40A	-	
EAA05204	543		3' truncated	3L 42A	-	
EAA13743	187		full	3L 40A	-	
EAA01418	362	337	3' truncated	2R 10A	-	
EAA05160	216		3' truncated	3L 42B	+	NAH, IRB, Blood1
EAA04072	280	258	full	2L 26B	+	NAH, blood1
EAL39349	262		3' truncated	3L 40A	-	
EAA05042	777	756	full	3L 42A	+	Blood1, cDNA1
EAA03931	178		full	2L 26D	+	Blood1, cDNA1, NAH
EAA02818	144		3' truncated	Unknown	+	NAP1
EAA09906	171		5' truncated	3L 39A	+	NAH, NAFB, Blood1
EAL39350	330	308	full	3L 40A	-	
EAL39343	284		3' truncated	3L 40A	-	
EAA13689	178		3' truncated	3L 40A	-	
EAA04169	234		3' truncated	2L 26A	-	
EAL41889	339		full	2L 26D	-	
EAA05087	211		3' truncated	3L 42A	-	
EAA06922	323	267	3' truncated	X 5A	+	NAH, NAFB, Blood1
EAA01294	185		full	2R 8C	-	
EAA15009	183		5' truncated	3R 33B	+	NAP1
EAL39347	242		3' truncated	3L 40A	-	
EAA13749	180		3' truncated	3L 40A	-	
EAA05439	266		3' truncated	3L 40B	-	
EAA05095	259	230	3' truncated	3L 42A	-	
AAR01125	268		3' truncated	Unknown	-	
EAA13688	1020		3' truncated	3L 40A	+	cDNA1
EAA05097	166		3' truncated	3L 42A	-	
EAL39030	81		3' truncated	3R 33B	+	NAP1
EAA05065	116		3' truncated	3L 42A	-	
EAL40630	94		3' truncated	Unknown	-	
EAA13692	441		Full	3L	+	NAP1
EAA02970	321	300	Full	Unknown	-	
EAA13755	596		Full	3L	-	
EAA13691	231		Full	3L 40A	-	
EAA13726	212		Full	3L 40A	+	NAFB
EAA13760	271		Full	3L	+	cDNA1
EAA10480	284	265	Full	2L	-	
EAA05069	227	204	3' truncated	3L	+	NAP1
***D. melanogaster***						
AAM68209	291	271	Full	2R 58B9	+	GH
AAF57948	246	225	Full	2R 53D1	+	RE
AAF44911	187	167	3' truncated	2L 34C4	-	
AAF59068	347		Full	2R 44D4	-	
AAF52372	176		5' truncated	2L 26C3	-	
AAF48780	358	335	Full	X 16F1	+	LP
AAM52597	195		Full	X 9A3	+	RE, GH
AAF46536	332	310	Full	X 9A3	+	RH, GH, EK
AAN09619	241		Full	X 9A3	+	RH, GH, EK
AAL48972	198	177	3' truncated	2R 53D1	+	RE
AAF47782	459	436	Full	3L 63E5	+	RE, GM, EK,LP,CA
AAF58455	799	758	Full	2R 49D3	+	RE, SD,RE,EK,LP
AAF55227	363		Full	3R 89A5	+	
AAF49079	422		Full	3L 76E1	+	RE, GM, EK, EC
AAN11645	406		Full	3L 76E1	+	EK, GM
AAM11109	154		5' truncated	3L 76E1	+	EK, GM
AAF46535	334	315	Full	X 9A3	+	RE, GH
AAN09447	251		Full	X 16F1	+	LP
AAF46801	157		5' truncated	2R 58B8	-	
AAA28880	774	752	Full	2R 49D3	+	RE, SD,RE,EK,LP

1. P represents precursor form of the predicted protein. M represents mature form of the predicted protein. Blank in column M indicates that signal is unpredictable.

2. FBG domain classified three categories. Full is the protein containing entire FBG domain; 5' truncated is the protein containing part of FBG domain which is truncated at the 5 primer region; 3' truncated is the protein containing part of FBG domain which is truncated at the 3 primer region.

3. In the transcription, + indicates matched transcript in EST database, – indicates no matched transcript in EST database. Tissue distribution was represented with the short-written of the EST library that was described in table 2.

**Figure 1:**
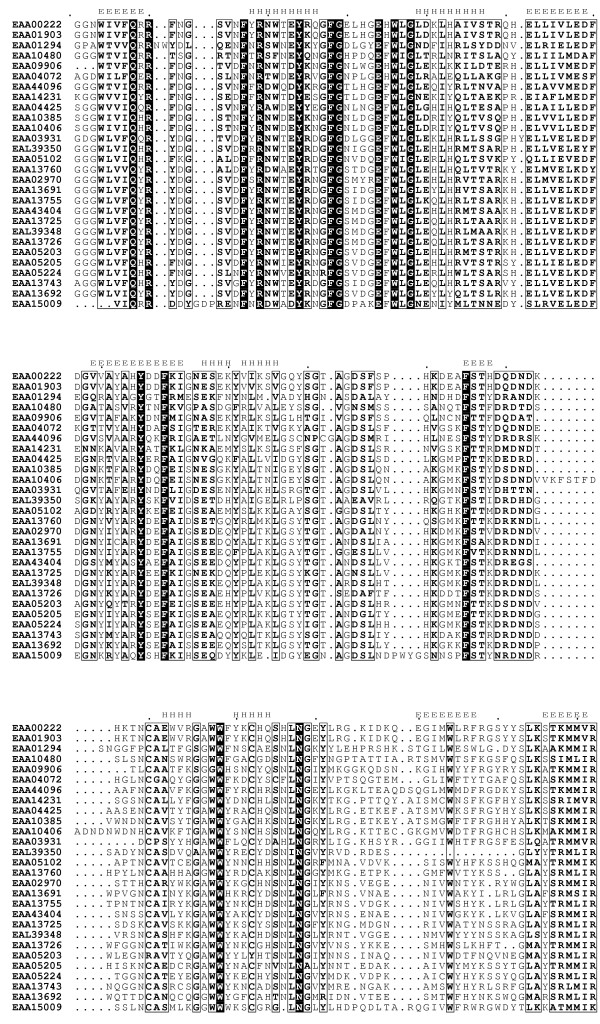
**Multiple sequence alignment of a representative set of the FBG domains of the FREP family in *A. gambiae*. **Multiple sequence alignment was constructed using T-Coffee program. The 100% consensus sequence was boxed with black in the alignment. The PHD secondary structure is shown above the alignment with H representing an α-helix and E representing a β-strand. The sequences are denoted by their gene names in GenBank.

To define the FBG domain in the FREP family, all 53 FREP and the human fibrinogen chain γ were aligned with the T_Coffee program. The results showed that most of the FREP genes have a C-terminal region composed of approximately 200aa with high sequence similarity with the C-terminus of human fibrinogen chain γ. Based on the alignment, the highly conserved region of 200aa residues in FREP was defined as the FBG domain in this study. A selected number of the FBG domains of the FREP were aligned and the highly conserved regions are illustrated in Fig. 1. This definition also is supported by the FBG domains in human and mouse ficolins [2]. In the FREP gene family, 28 of the 53 FREP genes were found in complete open reading frames and with a full FBG domain, and the remaining 25 FREP genes have truncated FBG domains, either in the 5'-region or the 3'-region (Table 1). Using a signal peptide prediction program, 14 of the 53 FREPs were predicted to contain secretion signal peptides (Table 1), suggesting that FREPs can be extracellular or intracellular.

### Conserved structure of the FBG domain in the FREP family and variation in some members

**Figure 2:**
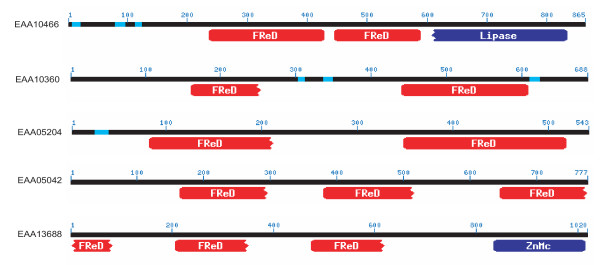
**Distribution of multiple FBG domains in the members of FREP family in *A. gambiae*. **The protein is represented by a line with the number above corresponding to amino acids which start from the N-terminus of each protein. The identified domains are shown under the line. FReD represents FBG domain. ZnMc represents Zinc-dependent metalloprotease domain. The sequences are denoted by their gene name in GenBank.

To construct an optimal multiple alignment of the FBG domain, we first aligned selected sequences with the T_Coffee program; this was followed by refinements on the basis of the PSI-BLAST search results. The selected multiple sequence alignment is shown in Fig. 1. The multiple alignment of the FBG domain sequences shows that FBG domains are highly similar throughout. Strikingly, 53% (28/53) of the FREPs contained a full FBG domain in their C-terminus (Table 1). Interestingly, some of the FREPs contain more than one FBG domain, although most of them are all not full FBG domains (Fig. 1). The distribution of the multiple FBG domains in these proteins shows certain patterns. Some of them contain two FBG domains that are connected by a 150aa hinge, e.g., EAA10360 and EAA05204. However, the two FBG domains in EAA10466 are located in the center of the protein, and are hinged together by approximately 20aa residues. There are also some members that contain 3 FBG domains. In EAA05042, three equivalent length regions of the FBG domain were repeated in the sequence (Fig. 2). Some of the FREPs also are composed of other domains in addition to the FBG domain, such as Lipase in EAA10466 (Fig. 2). In invertebrates, several FREP proteins have been reported to play an important role in innate immunity and in particular in the recognition of parasites (TL5A, AL-1). AL-1 can be upregulated by bacterial challenge and is able to bind GlcNAc and bacteria [13]. The FBG domain of TL5A can form a ligand-binding pocket specifically recognizing the acetyl-group in eliciting an immune response [16]. These data suggest that the FBG domains of FREPs probably function in recognizing carbohydrate moieties as part of the role they play in the mosquito immune response.

**Figure 3:**
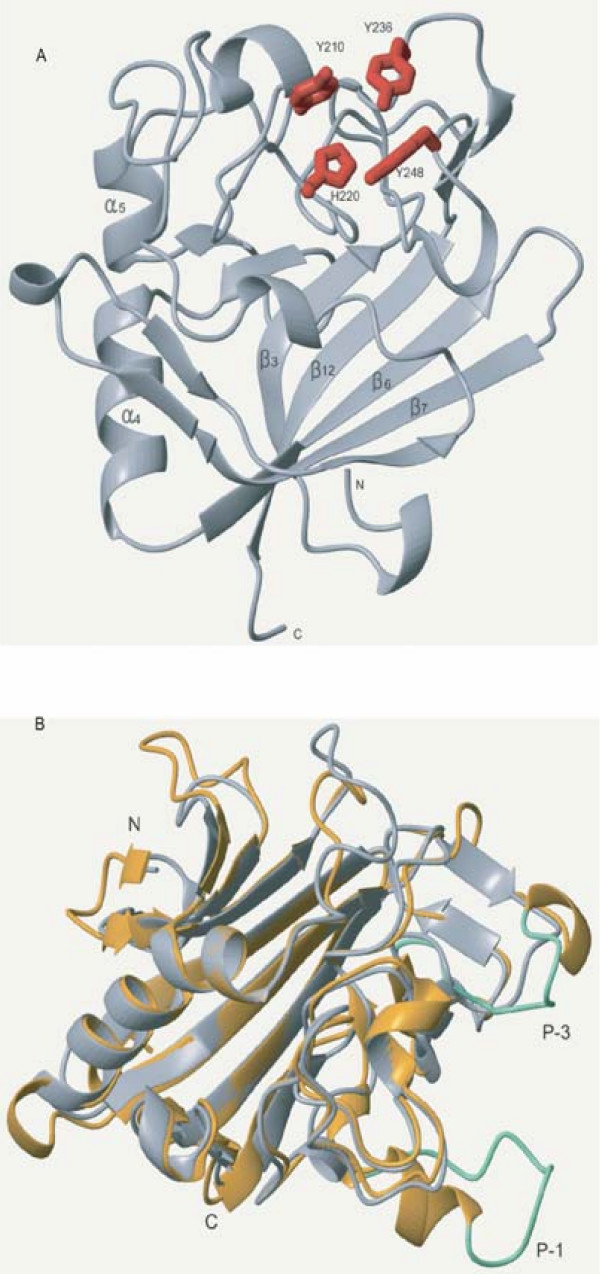
**Ribbon representation of the core structure of the FBG domain of tachylectin 5A (PDB: 1JC9) and recombinant human γ-fibrinogen carboxyl terminal fragment (PDB: 2FIB). **A. Ribbon plot of the FBG domain of TL5A. The domain shown here is a cartoon representation from the crystal structure. Main α-helices and β-sheets were shown in the figure. The residues forming the ligand-binding packet are depicted in the stick format and labeled in red. B. Superposition of the crystal structure of the FBG domain of TL5A (grey) and human γ-fibrinogen carboxyl terminal fragment (golden). By aligning TL5A and the γ chain fragment, the region composed of 178aa residues at the C-terminal regions of both proteins was used to generate superposition ribbon plot. Loop P-1 and P-3 in fibrinogen γ chain fragment are represented in green.

**Figure 4:**
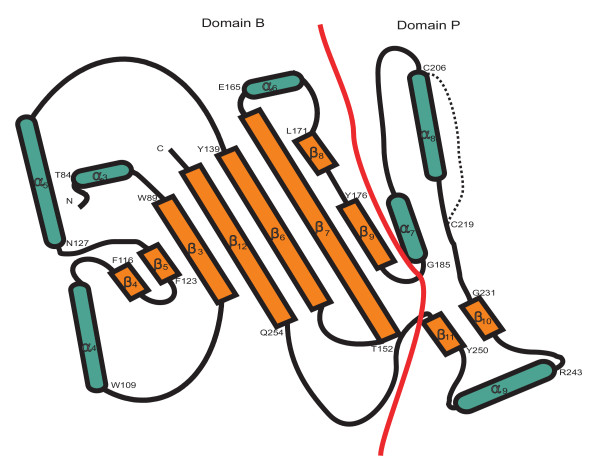
**Topology diagram showing the arrangement of secondary-structure elements in the FBG domains of TL 5A. **Domains named in analogy to human fibrinogen γ chain fragment. α-helix is represented in green and β-sheet is represented in brown. Domain B and domain P are separated by a red line. Starting position of amino acid in each secondary structure is shown in the figure with single letter. The disulfide bridge (Cys-206-Cys-219) in the domain P is represented by a dot line.

Using the multiple alignment of the FBG domains as queries, the secondary structure was predicted with the PHD program. The results show that the FBG domains have a highly conserved structure profile throughout the FBG domain (Fig. 1). By comparison of the predicted secondary structure with multiple alignment, most of these secondary structures fall in the conserved region, suggesting that FBG domains have similar domain architectures in the FREP gene family. To further compare the predicted secondary structure of the FBG domains with known structures, we found that the FBG domain is structurally related to the human fibrinogen γ fragment and the FBG domain of TL5A in the protein data bank (PDB) (Fig. 3A and 3B) [16,17]. The FBG domains of human fibrinogen γ fragment and TL5A compose the central and larger domain B and a relatively smaller domain P (Fig. 4). The domain B is predominantly built up by a twisted seven-stranded antiparallel β-sheet (strands β3-β7, β9 and β12) and helices α4 and α5 (Fig. 3A), and their tertiary structure is very similar (Fig. 3B). The domain P possesses only a few short elements of secondary structure, and comprises the major functional site forming a binding pocket [16]. The predicted secondary structures of the FBG domains in the FREP gene family approximately correspond to the domain architectures of FBG domains in human fibrinogen γ chain and TL5A. The β-sheets and α-helices in the predicted structure of the FBG domain are highly conserved with the corresponding structures in TL5A, especially in the domain B (Fig. 1 and Fig. 3B). For example, the central strand β12, which extends the C terminus of domain P back to domain B and brings both polypeptide termini in close proximity, was also seen in the FBG domains (Fig. 1 and Fig. 4). This suggests that the FBG domain architecture is conserved between houseshoe crab and mosquitoes. The projection of some of the highly conserved domains that form the ligand-binding pocket suggests that the core structure of the ligand-binding pocket is also likely to be conserved across these FBG domains (Fig. 1, Fig. 3B and Fig. 4). These observations imply that the FBG domains are most likely to function as receptors for carbohydrates or their derivatives. Beyond the common core, FBG domains also show great diversity in terms of the insertions and deletions among the conserved domains. Some FBG domains lose a conserved domain due to deletion, such as EAL39350. Other members have a short insertion located in the loop region, such as EAA10406 and EAA15009 (Fig. 1). By comparison of amino acids in the FBG domains of FREP corresponding to the P domain binding site in TL5A, we found that the domain architectures of these FBG domains have considerable diversity that is incorporated into a shared basic architectural blueprint (Fig. 1).

**Figure 5:**
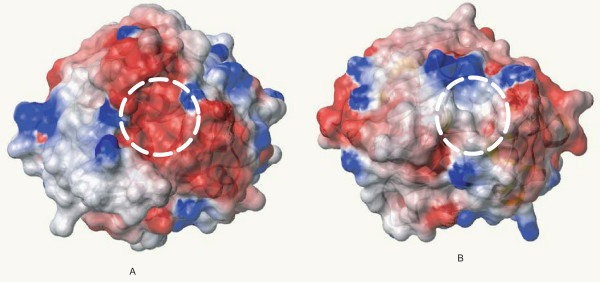
**Recombinant human γ-fibrinogen carboxyl terminal fragment (A) and surface of electrostatic potential of tachylectin 5A (B). **A. Negative charged patch was outlined in circle. B. Hydrophobic groove was outlined in circle. The orientation is the same in both A and B. Red is for negative charge, blue is for positive charge and grey is non-polar areas.

In domain P of TL5A, a disulfide bridge Cys-206-Cys-219 is an important structure to connect the metal-binding site to the acetyl group recognition site. These two conserved cysteines were seen in the FBG domains of the FREP family (Fig. 1). Furthermore, four aromatic side chains (Tyr-210, Tyr-236, Tyr-248, and His-220), which can form a funnel to the acetyl-group in TL5A, were also seen in most of the FBG domains of FREPs (Fig. 1). In some of the FBG domains, the amino acids corresponding to the binding sites have mutated. This great diversity probably provides the variability necessary for these FBG domains to form slightly different binding sites that could recognize different carbohydrates. This provides a diverse and potential flexible arsenal for the host to recognize a variety of correspondingly diverse carbohydrates on the surface of pathogens. Alternatively, it is likely that some of the FBG domains have other unknown functions besides recognition. Beyond the conservation of the full FBG domains in the FREP gene family, FBG domains show great variety in terms of their lengths. Multiple sequence alignment shows that 24 of the 53 FREPs consist of truncated FBG domains (data not shown). Multiple sequence alignment shows that 24 of the 53 FREPs consist of truncated FBG domains (Table 1). The lengths vary from 30 to 160 amino acids. Many of them are truncated in the C-terminus. By scanning the corresponding genome sequences using Artemis, we found some of the truncated parts of the FBG domain exist in the genome in close relation to the annotated fragment, suggesting that the truncation probably was a missannotation of the genome. By comparison of sequence similarity and structural profile, the recognition sites in the FBG domains of FREP and TL5A correspond structurally to the polymerization pocket in the fibrinogen γ fragment (Fig. 3A and 3B). Five of the seven amino acids that form the polymerization pocket are structurally equivalent to amino acids in the sugar-binding site of TL5A. The long loops P-1 and P-3 in the fibrinogen γ fragment are shortened by 14 and 7 amino acids respectively in the FBG domains and TL5A, and represent the major structural differences found in the functionally important domain P. The domain P also has very different surface charge in the two structures. On 1FIB, it forms a highly negative charged patch (Fig. 5A), while it is mainly hydrophobic on 1JC9 (Fig. 5B), which probably contributed to their target specificities. Variability in this domain points to a potential evolutionary transition from a carbohydrate to a protein-binding module [16,17].

### Phylogenetic relationships of the FBG domains in *A. gambiae*

**Figure 6:**
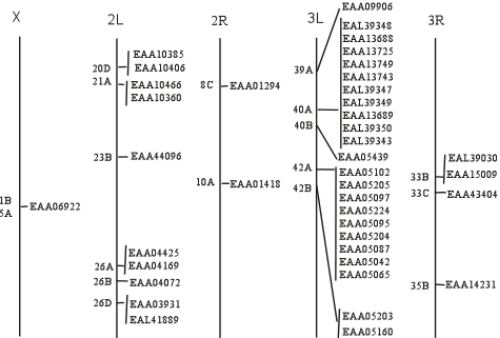
**Genomic distribution of FREP family members in *A. gambiae*. **Chromosomes are represented with a line and chromosomal numbers are shown on the top of each chromosome. Chromosomal loci of the FREP genes are shown with their name. The proteins are denoted by their gene name in GenBank.

To understand the evolutionary history of this gene family, an attempt was made to identify correlations between chromosomal locations of FREP and FBG domain sequence similarities among the family members. The genes for the FREP family in *A. gambiae *have been mapped to specific *A. gambiae *chromosomal locations by retrieving Locuslink from Ensembl. Of the 53 FREP genes, chromosomal locations could be determined for (Fig. 6). The majority of FREP genes are found in clusters on chromosomes 2L and 3L, and some of these genes are arrayed in tandem. Twenty three genes located on chromosome 3L form 2 large clusters and 10 genes located on the chromosome 2L form two small clusters. This suggests that the FREP gene family evolved by expansion. FBG domains tandemly linked present a target for mispairing and unequal crossover, which could have resulted in duplication and divergence of the genes over time. These tandemly duplicated FBG domains could then become physically separated through chromosomal rearrangements and translocation. This suggests a dynamic history for the FBG domains that is likely to have involved gene expansion, with the FREP gene family evolving through vast expansion of the FBG domain.

**Figure 7:**
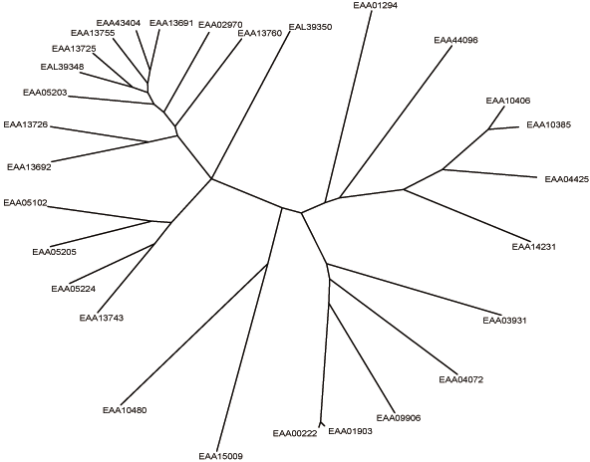
**Phylogenetic tree of the FBG domains of the FREP family in *A. gambiae*. **Phylogenetic relationships of the FBG domains are shown. The seed alignment used for constructing the tree was the multiple alignment sequences shown in Fig. 1. Maximum-likelihood approach was used to construct the tree with the proml program of the PHYLIP package, which uses the Jones-Taylor-Thornton model of change between amino acids and a Hidden Markov Model (HMM) method of inferring different rates of evolution at different amino acid positions. The FBG domains of each FREP are denoted by their gene name in GenBank.

To analyze the evolutionary history of FBG domains in the FREP family, a phylogenetic tree was constructed with the alignments of the conserved FBG domains using maximum-likelihood methods (Fig. 7). This tree showed that the FBG domains were grouped into several branches. However, a major branch was observed in the evolutionary tree of the FBG domains. This branch is comprised largely of FBG domains of the FREP family from the *A. gambiae *genome. If the number of FBG domains increased mainly by tandem duplication, we would expect the domains which are physically clustered in the genome to form a monophyletic group. However, by examinating the relationships between phyletic pattern and chromosomal location of the FBG domains, it is found that some FBG domains grouped together in the phylogenetic tree are located on different chromosomes, such as EAA09906 and EAA04072, EAA43404 and EAA13725 (Fig. 6 and Fig. 7). This suggests that a dynamic history for the FBG domains likely involved shuffling among chromosomes. The predicted role, for at least a subset of these FBG domains, is in carbohydrate sensing. This expansion in the *A. gambiae *genome may have been a response to the diversity of carbohydrates encountered, resulting in the utilization of numerous FBG domain variations in order to recognize a broad range of different carbohydrates.

### ESTs for FREPs in mosquitoes

To confirm that the conceptual FREP proteins predicted from the genome are actually transcribed in mosquitoes, we searched the *A. anopheles *EST database. Twenty one of the 53 predicted genes were identified to have transcripts (Table 1). Examination of the transcript resources reveals that these genes are likely to be expressed in different tissues in mosquitoes, such as fat body, midgut and head (Table 1 and 2). Some of these genes also are expressed following immune challenges and a blood meal. These results suggest that FREP genes probably play a role in immune responses or any of the diverse array of physiologies associated with blood feeding. However, more than 50% of the predicted FREP genes have not been identified transcriptionally in the EST database. It is possible that the EST database does not cover the entire transcriptome and greater coverage is needed. To compare the actual transcripts of the FREP genes in different mosquito species, the FREP transcripts in *Ar. subalbatus *and *Aedes aegypti *were searched in the immune challenged hemocyte EST databases at ASAP [18]. Five and 12 different genes were transcribed respectively in the bacteria-challenged hemocytes. This suggests that some of the FREP genes are hemocyte-associated and possibly involved in innate immune responses post bacteria inoculation [19].

**Table 2:** Description of EST libraries from *A. gambiae *and *D. melanogaster*

**Name**	**Description**	**Supplier**
***A. gambiae***		
NAP1	mix developmental stages	European Molecular Biology
NAFB	Normalized Fat Body Library	University of Notre Dame
cDNA1	Adult cDNA1	Celera Genomics
4A3B	cDNA libraries derived from immune-responsive hemocyte-like cell lines	
blood1	Adult with blood-fed cDNA	Celera Genomics
NAH	Normalized Anopheles Head	University of Notre Dame
IRB	Infected Rat Blood-fed 30 hr Abdomen, Female adult 5–7 days post eclosion	University of Notre Dame
		
***D. melanogaster***		
GH	Adult male and female head	
RE	normalized Embryo from male and female, 0–24 hours mixed stage embryonic	Lawrence Berkeley National lab
LP	Whole body Larval-early pupal from male and female	
RH	Adult male and female normalized Head pFlc-1	Lawrence Berkeley National lab
EK	Mixed stage embryos, imaginal disks and adult head	Lawrence Berkeley National lab
GM	Ovary, newly eclosed females, germarium-stage 6, female.	
SD	Schneider L2 cell culture pOT2, cell line	British Columbia Cancer A
CA	Male and female salivary gland, 16, 18, 20, 22, and 24 hrs after puparium formation	
EC	Fat body-3rd instar larva	Lawrence Berkeley National lab

### Fibrinogen-related proteins in *D. melanogaster*

**Figure 8:**
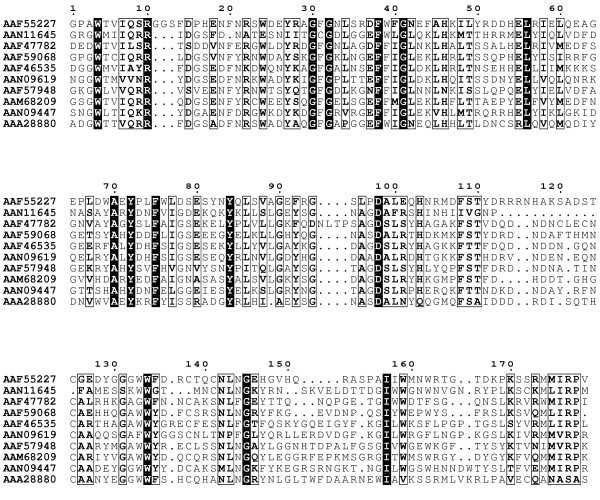
**Multiple sequence alignment of a representative set of the FBG domains of FREP in *D. melanogaster*. **Multiple sequence alignment was constructed using T-Coffee program. The 100% consensus sequence was boxed with black in the alignment. The PHD secondary structure is shown above the alignment with H representing an α-helix and E representing a β-strand. The sequences are denoted by their gene name in GenBank.

*D. melanogaster *is an important experimental insect and is used as a standard research model in the biomedical sciences. *D. melanogaster *is closely related to mosquitoes, with both insects belonging to the order Diptera. To compare the evolutionary development of the FBG domains between mosquito and fruitfly, detailed analyses of conserved segments were conducted. By searching the NCBI database, 20 FREP conceptual proteins were predicted in the *D. melanogaster *genome (Table 1). The multiple alignment of the FBG domain sequences showed that conservation exists throughout the FBG domain region (Fig. 8). Truncated FBG domains also exist in FREPs in *D. melanogaster *(Table 1). For example, two members of the FREP gene family have 3'-truncated FBG domains (AAF44911 and AAL48972). To further understand the relationships of the FBG domains between *A. gambiae *and *D. melanogaster*, a phylogenetic tree was constructed by using the conserved FBG domains from both species. The most striking pattern observed in the evolutionary tree was the presence of multiple branches comprised largely of proteins from a single organism (Fig. 10). These lineage specific expansions accounted for most of the FBG domains in *A. gambiae *and *D. melanogaster*. Furthermore, a branch comprised of the FBG domains from both *A. gambiae *and *D. melanogaster *was also noted (EAA01294, EEA15009, AAF55227, AAA28880) (Fig. 10).

**Figure 9:**
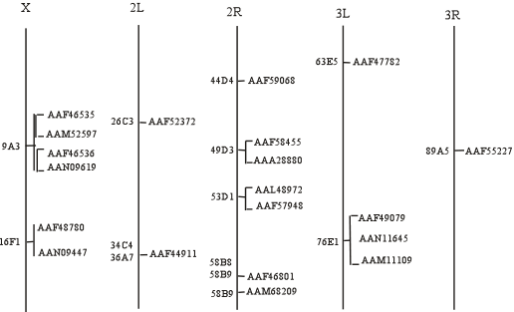
**Genomic distribution of FREP family members in *D. melanogaster*. **Alternative spliced transcripts from the same gene are represented with [. The others are as detailed in Figure 6.

To determine genomic distribution of FREP members, the chromosomal location for every sequence was found by using the Locuslink program. Position information showed that some genes have more than one transcript (Fig. 9). To get the detailed information about these genes, a comparison of mRNA and genome sequences was performed by using the Spidey program at NCBI. The results showed that the predicted proteins from the same genes are generated by alternative splicing among exons and introns post transcription. Some of the FBG domains come from the same transcription region, such as AAF46535 and AAM52597. This would generate the same FBG domains. However, some of the FBG domains are generated from different regions. For example, transcription of the FBG domain in AAN09447 is located in a big intron between the first two exons in AAF48780, resulting in different FBG domains. To determine the actual fully processed transcripts of these genes, a search of the EST database was conducted. Thirteen of the 20 FREP proteins were identified in the *D. melanogaster *transcript database. By examining the genomic location, we found that AAF49079, AAN11645 and AAM11109 are transcribed from the same gene. The actual transcripts of these 3 gene products are also represented in the EST database. This further illustrates the complexity of gene regulation post transcription, which could provide multiple protein products from a single gene, thereby, further increasing variation in the FREP family.

**Figure 10:**
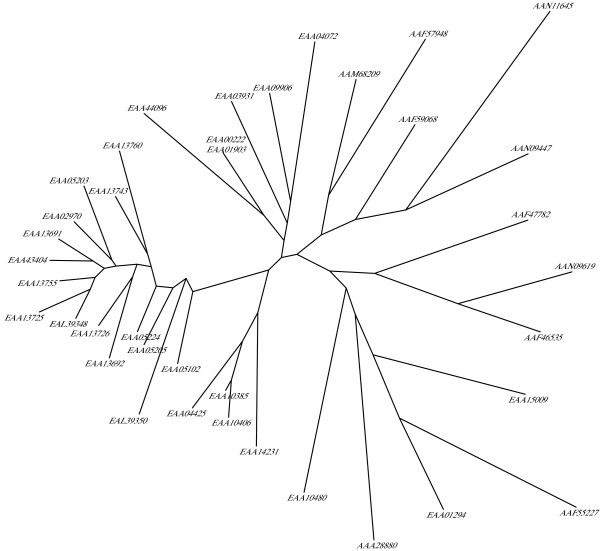
**Phylogenitic tree of the FBG domains from *A. gambiae *and *D. melanogaster*. **The seed alignment used for constructing the tree was the multiple alignment sequences of representative set of the FBG domains of FREP families in *A. gambiae *and *D. melanogaster*. The phylogenetic tree was constructed as described in methods and detailed in Fig. 5. The FBG domains of each FREP are denoted by their gene name in GenBank. The name of the FREP from *A. gambiae *start with E, and the name of the FREP from *D. melanogaster *start with A.

Compared with the *D. melanogaster *FREP gene family, the massive expansion of the FREP gene family in mosquitoes probably is associated with particular aspects of the mosquito's biology, possibly hematophagy and exposure to parasites [15]. The blood meal imposes challenges associated with proliferation of the microbial flora in the gut and coagulation of ingested blood and penetration of the midgut by blood-born pathogens. A FREP protein (e.g AL-1) in the mosquito *Ar. subalbatus *has bacteria binding properties, and it has been suggested that FREP may be important in controlling bacteria infections in mosquitoes [13]. However, mosquitoes may use a number of FREP proteins as anticoagulants, for instance, as competitive inhibitors preventing polymerization of blood [15]. Some mosquito FREP genes are up-regulated by invading malaria parasites [20,21], suggesting a possible role in an antimalarial defense system.

## Conclusion

The detailed sequence and structural analyses disclose that the FREP family contains highly similar FBG domains in the *A. gambiae *genome. FBG domains are predicted to recognize carbohydrates and their derivatives. The sequence divergence seen in the binding domains of FBG domains makes it possible to recognize a wide range of carbohydrate derivatives. This suggests that the FREP family may play an important role in innate immunity. Expansion of the family during evolutionary history is mainly accounted for by a major expansion of the FBG domain architectures. Further analysis of the chromosomal locations and phyletic patterns of the FBG domains suggest that they have been acquired by tandem duplication and shuffling. Compared with *D. melanogaster*, the massive expansion of the FREP family in *A. gambiae *probably is associated with particular aspects of the mosquito's biology, such as exposure to parasites and hematophagy. Experimental investigations of these proteins are likely to be of interest in understanding insect innate immunity and physiology.

### Methods

#### Database searching and sequence retrieving for fibrinogen-related protein

A PSI-BLAST search [22] of the *A. gambiae *and *D. melanogaster *genome database at the National Center for Biotechnology Information (NCBI) [23] was performed using AL-1 as a query. To obtain the recent progress of FREP in *A. gambiae *genome, the *A. gambiae *database at Ensembl [24] was also searched. Following accumulation of the complete list of accession numbers, the corresponding protein sequence was retrieved from GenBank at NCBI and Ensembl.

### Signal peptide prediction

Signal peptides were predicted using the SignalPv3.0 [25,26].

### Searching for ESTs database

To determine the actual transcripts for individual FREP genes, BLAST search of an EST database at Berkeley *Drosophila *Genome Project and TIGR *A. gambiae *Gene Index (AgGI) was performed [27,28]. The annotated cDNA sequences encoding FREPs identified in the PSI-BLAST search were used as queries for individual BLAST search in these EST database. The availability of EST was determined based on sequence similarity with the query: a 97% or greater identity was considered to be an EST corresponding to a specific gene. To get information about FREP transcripts in the mosquito, *Ar. Subalbatus *and *Ae. aegypti*, hemocyte EST databases at ASAP in both species were searched using AL-1 as a seed [18,29].

### Multiple sequence alignment and phylogenetic analysis

Multiple sequence alignment was performed using the T-Coffee program [30,31]. Phylogenetic analysis was carried out with the maximum-likelihood algorithm [32]. The package used for phylogenetic analysis was proml program from PHYLIP [33], and the unrooted tree was draw using drawtree program in this package.

### View of DNA sequence annotation

To verify the annotation of truncated genes, the corresponding genomic sequences was scanned by Artemis [34].

### Secondary structure prediction

Secondary structure prediction was produced with the PHD program [35], with multiple alignment of individual FBG domains of FREP family. The structure data of TL5A and recombinant human γ-fibrinogen carboxyl terminal fragment were obtained from protein data bank (PBD) [36] and the ribbon diagrams were constructed with Molmol program [37].

### Chromosomal location and alternative splice transcripts

The chromosomal location of the FREP genes in *A. gambiae *genome was retrieved at Ensembl [24]. The chromosomal location of the FREP genes in *D. melanogaster *was retrieved at NCBI [23]. To identify alternative spliced transcripts for each gene, spidey, a cDNA-to-genomic alignment program, was used to align spliced sequences to genomic sequences, using local alignment algorithms and heuristics to put together a global spliced alignment [38].

## Abbreviations

FBG domain, fibrinogen-like domain; FREP, fibrinogen-related protein; AL-1, aslectin, TL5A, tachylectin 5A; GlcNAc, *N*-acetylglucosamine; MAP, microfibril-associated protein; aa, amino acid; BLAST, basic local alignment search tool; PSI-BLAST, position specific iterative BLAST; EST, expressed sequence tag; PDB, protein data bank; Molmol, molecule analysis and molecule display.

## Authors' contributions

XW carried out the database survey. He identified and analyzed the FBG domains, and prepared the manuscript. QZ generated ribbon diagram and did structure analyses. BMC conceived the study and contributed to the preparation of the manuscript. All authors read and approved the final manuscript.

## References

[B1] Gorkun OV, Veklich YI, Weisel JW, Lord ST (1997). The conversion of fibrinogen to fibrin: recombinant fibrinogen typifies plasma fibrinogen. Blood.

[B2] Lu J, Le Y (1998). Ficolins and the fibrinogen-like domain. Immunobiology.

[B3] Matsushita M, Fujita T (2002). The role of ficolins in innate immunity. Immunobiology.

[B4] Erickson HP (1993). Tenascin-C, tenascin-R and tenascin-X: a family of talented proteins in search of functions. Curr Opin Cell Biol.

[B5] Kobayashi R, Mizutani A, Hidaka H (1994). Isolation and characterization of a 36-kDa microfibril-associated glycoprotein by the newly synthesized isoquinolinesulfonamide affinity chromatography. Biochem Biophys Res Commun.

[B6] Lu J, Teh C, Kishore U, Reid KB (2002). Collectins and ficolins: sugar pattern recognition molecules of the mammalian innate immune system. Biochim Biophys Acta.

[B7] Teh C, Le Y, Lee SH, Lu J (2000). M-ficolin is expressed on monocytes and is a lectin binding to N-acetyl-D-glucosamine and mediates monocyte adhesion and phagocytosis of Escherichia coli. Immunology.

[B8] Zhao Z, Lee CC, Jiralerspong S, Juyal RC, Lu F, Baldini A, Greenberg F, Caskey CT, Patel PI (1995). The gene for a human microfibril-associated glycoprotein is commonly deleted in Smith-Magenis syndrome patients. Hum Mol Genet.

[B9] Gokudan S, Muta T, Tsuda R, Koori K, Kawahara T, Seki N, Mizunoe Y, Wai SN, Iwanaga S, Kawabata S (1999). Horseshoe crab acetyl group-recognizing lectins involved in innate immunity are structurally related to fibrinogen. Proc Natl Acad Sci USA.

[B10] Adema CM, Hertel LA, Miller RD, Loker ES (1997). A family of fibrinogen-related proteins that precipitates parasite-derived molecules is produced by an invertebrate after infection. Proc Natl Acad Sci USA.

[B11] Kenjo A, Takahashi M, Matsushita M, Endo Y, Nakata M, Mizuochi T, Fujita T (2001). Cloning and characterization of novel ficolins from the solitary ascidian, *Halocynthia roretzi*. J Biol Chem.

[B12] Schroder HC, Ushijima H, Krasko A, Gamulin V, Thakur NL, Diehl-Seifert B, Muller IM, Muller WE (2003). Emergence and disappearance of an immune molecule, an antimicrobial lectin, in basal metazoa. A tachylectin-related protein in the sponge *Suberites domuncula*. J Biol Chem.

[B13] Wang X, Rocheleau TA, Fuchs JF, Hillyer JF, Chen CC, Christensen BM (2004). A novel lectin with a fibrinogen-like domain and its potential involvement in the innate immune response of *Armigeres subalbatus *against bacteria. Insect Mol Biol.

[B14] Redfern O, Grant A, Maibaum M, Orengo C (2005). Survey of current protein family databases and their application in comparative, structural and functional genomics. J Chromatogr B Analyt Technol Biomed Life Sci.

[B15] Zdobnov EM, von Mering C, Letunic I, Torrents D, Suyama M, Copley RR, Christophides GK, Thomasova D, Holt RA, Subramanian GM, Mueller HM, Dimopoulos G, Law JH, Wells MA, Birney E, Charlab R, Halpern AL, Kokoza E, Kraft CL, Lai Z, Lewis S, Louis C, Barillas-Mury C, Nusskern D, Rubin GM, Salzberg SL, Sutton GG, Topalis P, Wides R, Wincker P, Yandell M, Collins FH, Ribeiro J, Gelbart WM, Kafatos FC, Bork P (2002). Comparative genome and proteome analysis of *Anopheles gambiae *and *Drosophila melanogaster*. Science.

[B16] Kairies N, Beisel HG, Fuentes-Prior P, Tsuda R, Muta T, Iwanaga S, Bode W, Huber R, Kawabata S (2001). The 2.0-A crystal structure of tachylectin 5A provides evidence for the common origin of the innate immunity and the blood coagulation systems. Proc Natl Acad Sci USA.

[B17] Yee VC, Pratt KP, Cote HC, Trong IL, Chung DW, Davie EW, Stenkamp RE, Teller DC (1997). Crystal structure of a 30 kDa C-terminal fragment from the gamma chain of human fibrinogen. Structure.

[B18] Glasner JD, Liss P, Plunkett G, Darling A, Prasad T, Rusch M, Byrnes A, Gilson M, Biehl B, Blattner FR, Perna NT (2003). ASAP, a systematic annotation package for community analysis of genomes. Nucleic Acids Res.

[B19] Bartholomay LC, Cho WL, Rocheleau TA, Boyle JP, Beck ET, Fuchs JF, Liss P, Rusch M, Butler KM, Wu RC, Lin SP, Kuo HY, Tsao IY, Huang CY, Liu TT, Hsiao KJ, Tsai SF, Yang UC, Nappi AJ, Perna NT, Chen CC, Christensen BM (2004). Description of the transcriptomes of immune response-activated hemocytes from the mosquito vectors *Aedes aegypti *and *Armigeres subalbatus*. Infect Immun.

[B20] Dimopoulos G, Christophides GK, Meister S, Schultz J, White KP, Barillas-Mury C, Kafatos FC (2002). Genome expression analysis of *Anopheles gambiae *: responses to injury, bacterial challenge, and malaria infection. Proc Natl Acad Sci USA.

[B21] Srinivasan P, Abraham EG, Ghosh AK, Valenzuela J, Ribeiro JM, Dimopoulos G, Kafatos FC, Adams JH, Fujioka H, Jacobs-Lorena M (2004). Analysis of the *Plasmodium *and *Anopheles *transcriptomes during oocyst differentiation. J Biol Chem.

[B22] Altschul SF, Madden TL, Schaffer AA, Zhang J, Zhang Z, Miller W, Lipman DJ (1997). Gapped BLAST and PSI-BLAST: a new generation of protein database search programs. Nucleic Acids Res.

[B23] The National Center for Biotechnology Information (NCBI). http://www.ncbi.nlm.nih.gov..

[B24] The *A. gambiae *database at Ensembl. http://www.ensembl.org/Anopheles_gambiae/.

[B25] Nielsen H, Engelbrecht J, Brunak S, von Heijne G (1997). Identification of prokaryotic and eukaryotic signal peptides and prediction of their cleavage sites. Protein Engineering.

[B26] The SignalPv3.0. http://www.cbs.dtu.dk/services/SignalP/.

[B27] Berkeley *Drosophila *Genome Project. http://www.fruitfly.org/EST/.

[B28] TIGR *A. gambiae *Gene Index (AgGI). http://www.tigr.org/tdb/tgi/aggi/.

[B29] The mosquito, *Ar. Subalbatus *and *Ae. aegypti*, hemocyte EST databases at ASAP. https://asap.ahabs.wisc.edu/annotation/php/logon.php..

[B30] Notredame C, Higgins DG, Heringa J (2000). T-Coffee: A novel method for fast and accurate multiple sequence alignment. J Mol Biol.

[B31] The T-Coffee program. http://igs-server.cnrs-mrs.fr/Tcoffee/.

[B32] Felsenstein J (1996). Inferring phylogenies from protein sequences by parsimony, distance, and likelihood methods. Methods Enzymol.

[B33] PHYLIP. http://evolution.genetics.washington.edu/phylip.html.

[B34] Artemis. http://www.sanger.ac.uk/Software/Artemis/.

[B35] Rost B, Sander C (1993). Prediction of protein secondary structure at better than 70% accuracy. J Mol Biol.

[B36] Guex N, Peitsch MC (1997). SWISS-MODEL and the Swiss-PdbViewer: an environment for comparative protein modeling. Electrophoresis.

[B37] Koradi R, Billeter M, Wuthrich K (1996). MOLMOL: a program for display and analysis of macromolecular structures. J Mol Graph.

[B38] Spidey. http://www.ncbi.nlm.nih.gov/IEB/Research/Ostell/Spidey/spideywebeg.html.

